# Fecal Microbiota Transplantation: Screening and Selection to Choose the Optimal Donor

**DOI:** 10.3390/jcm9061757

**Published:** 2020-06-05

**Authors:** Stefano Bibbò, Carlo Romano Settanni, Serena Porcari, Enrico Bocchino, Gianluca Ianiro, Giovanni Cammarota, Antonio Gasbarrini

**Affiliations:** 1 UOC Medicina Interna e Gastroenterologia, Dipartimento di Scienze Mediche e Chirurgiche, Fondazione Policlinico Universitario “A. Gemelli” IRCCS, 00168 Roma, Italy; stefano.bibbo@policlinicogemelli.it (S.B.); carlosettanni@hotmail.it (C.R.S.); porcariserena89@gmail.com (S.P.); gianluca.ianiro@hotmail.it (G.I.); giovanni.cammarota@unicatt.it (G.C.); 2 Istituto di Patologia Speciale Medica, Università Cattolica del Sacro Cuore, 00168 Roma, Italy; enrico.bocchino01@gmail.com

**Keywords:** gut microbiota, precision medicine, *Clostridium difficile*, inflammatory bowel disease, ulcerative colitis, irritable bowel disease, metabolic syndrome

## Abstract

In the past decade, fecal microbiota transplantation (FMT) has rapidly spread worldwide in clinical practice as a highly effective treatment option against recurrent *Clostridioides difficile* infection. Moreover, new evidence also supports a role for FMT in other conditions, such as inflammatory bowel disease, functional gastrointestinal disorders, or metabolic disorders. Recently, some studies have identified specific microbial characteristics associated with clinical improvement after FMT, in different disorders, paving the way for a microbiota-based precision medicine approach. Moreover, donor screening has become increasingly more complex over years, along with standardization of FMT and the increasing number of stool banks. In this narrative review, we discuss most recent evidence on the screening and selection of the stool donor, with reference to recent studies that have identified specific microbiological features for clinical conditions such as *Clostridioides difficile* infection, irritable bowel syndrome, inflammatory bowel disease, and metabolic disorders.

## 1. Fecal Microbiota Transplantation: A New Old Therapy

In the last decade, multiple studies have expanded knowledge in the field of gut microbiota, including pathogenesis, diagnosis, and therapeutics [1]. To date, the therapeutic modulation of the intestinal microbiota is performed with traditional approaches such as antibiotics and probiotics, or increasingly through fecal microbiota transplantation (FMT), which is defined as the transfer of fecal material from a healthy donor into the gastrointestinal tract of a recipient [2].

Fecal material has been used in medicine since almost two thousand years. The first description of the use of fecal material for medical purposes dates back to about 1700 years ago; traditional Chinese medicine in particular had perceived the potential role of this biological material and used it for several clinical indications such as gastrointestinal, nervous system, skin, and gynecological diseases [3]. In Western countries, the first description of ancestral FMT dates to the 17th century, when Fabricius Acquapendente reported the transplantation of feces for the cure of animals unable to ruminate [4]. More recently, anecdotal use has been reported during the Second World War. German soldiers residing in North Africa suffered from recurrent episodes of diarrhea that they treated by eating camel stool, being inspired from the local practice of the Bedouins [5]. Western medicine began to study the potential role of FMT only in the second half of the 20th century. Firstly in 1958, Ben Eiseman reported the successfully treatment of four patients with pseudomembranous colitis using fecal enemas [6], and over 20 year later, Schwan et al. reported new evidence supporting the efficacy of FMT in *C. difficile* infection (CDI) [7]. In the following years, several other reports came out, and a growing body of evidence showed the efficacy of FMT in the treatment of recurrent CDI, and, furthermore, the feasibility of FMT was gradually suggested for other clinical indications.

The first randomized controlled trial that investigated the role of FMT for recurrent CDI was published by van Nood et al. in 2013. They reported that a single infusion of fecal material by nasoduodenal route was superior to standard therapy with vancomycin [8]. In further years, other routes of administration were successfully tested in clinical trials, demonstrating the efficacy of FMT by lower route through colonoscopy [9] or upper administration with capsule [10]. Therefore, the growing interest of the scientific community towards FMT has meant that a large amount of data has been published in the last decade; for this reason, a panel of European experts met in Rome in 2017 to release the first evidence-based consensus report for the use of FMT in clinical practice [11].

Over the years, further issues have emerged, which are still not clarified to date. In particular, in view of the growing number of patients who could benefit from FMT, it is necessary to identify innovative ways to storing fecal material to be used if necessary. Indeed, in the early experiences, FMT was performed only with fresh material from occasional healthy donors, but this approach is not feasible for large-scale use of FMT. To solve this problem, the possibility to create structures to bank the feces after manipulation was suggested, and this approach is supported by the evidence of the effectiveness of FMT performed with frozen material [12]. In consideration of the increasing interest of the scientific community on this topic, a panel of international experts met in Rome in 2019 to define the general guidelines for the creation of stool banks [13]. Despite these efforts, many problems remain to be solved. Above all, the identification of the optimal donor is a fundamental clinical issue of rising relevance. Indeed, the increasing number of clinical indications suggests the need to identify the ideal donor for each disease or patient that cannot be treated indiscriminately with the same fecal biomass. The fascinating idea of identifying the “perfect” intestinal microbiota has motivated the scientific community for at least one century—ever since in the early 20 century Elie Metchnikoff suggested the role of intestinal bacteria in the development of many pathological conditions and health in the homeostasis of the microbial species [14], generating the concepts of “eubiosis” and “dysbiosis,” which for years were considered only fascinating hypotheses without strong scientific bases. However, in recent years, the molecular techniques of genomic sequencing have allowed to understand the link between gut microbiota and several diseases [15], giving evidence to this old intuition. In particular, we refer to “eubiosis” as considering a status characterized by a preponderance of potentially beneficial species, while “dysbiosis” is a condition characterized by the loss of homeostasis and by the proliferation of microbial species considered potentially pathogenic and, moreover, favor a “milieu” triggering the hyper-inflammatory state [16]. To date, an increasing number of studies confirm these hypotheses, in particular the reduced diversity of gut microbiota, simply defined as the variety and abundance of species in a defined microbial ecosystem [17,18], which is known to characterize several chronic diseases compared to a control group [19,20]. Therefore, in this narrative review, we report the most recent evidences on the screening and selection of the stool donor, with special efforts to describe findings that may lead to the optimal donor in several disease looking for an “optimal microbiota” to be transplanted (CDI, inflammatory bowel disease (IBD), irritable bowel syndrome (IBS), and other emerging pathological conditions).

## 2. Fecal Microbiota Transplantation in Clinical Practice

To date, the only recommendation for FMT in clinical practice is the treatment of recurrent CDI, although a large number of emerging indications are being experienced in several studies [21].

CDI is a burdensome clinical issue and represent the most relevant cause of antibiotic-associated diarrhea; its incidence has evolved in recent years and the risk of recurrence after standard antibiotic therapy has widely increased [22,23]. The standard treatment for the first occurrence of CDI is still represented by antibiotic therapy, mainly with metronidazole or vancomycin [24]. However, the clinical success rate of antibiotics in the recurrence of CDI is dramatically decreased, consequently, more effective therapies have been proposed, including FMT [25,26]. The clinical success of FMT, in contrast to the loss of efficacy of standard antibiotic therapy, could be explained by understanding the mechanism of action. In fact, FMT is a restorative treatment of gut microbiota alterations, unlike antibiotics, which is a disruptive treatment; accordingly, the administration of FMT results in a prompt and sustained normalization of microbial community structure and then metabolic activity of gut microbiota [27]. Indeed, CDI develops only in subjects with disruption of gut microbiota [28]; supporting this idea, it was demonstrated that the feces of patients with recurrent CDI have a higher relative abundance of several bacterial family as *Enterobacteriaceae*, *Veillonellaceae*, and *Lactobacillaceae*, and lower relative abundance of *Ruminococcaceae*, *Bacteroidaceae*, and *Lachnospiraceae* [29]. Furthermore, FMT recipients have shown changes in microbial profiles and shifts in the gut microbiota composition towards a profile similar to that of the healthy donor; this finding is obtained in a few days and is observed for at least six months [30].

To date, several systematic review and meta-analyses have shown an overall cure rate of FMT of up to 90% in preventing further CDI recurrence [31,32]. Moreover, a recent meta-analysis has shown that both the upper and the lower route are effective, with a slight advantage of colonoscopy over other techniques [32]. Based on these positive evidences, scientific societies have included FMT among the recommended treatment for recurrent CDI. Already in 2014, FMT was strongly recommended in recurrent CDI by the European Society of Clinical Microbiology and Infectious Disease (ESCMID) [33], while the American College of Gastroenterology (ACG) stated that FMT can be considered after the third recurrence [34]; more recently, the Infectious Disease Society of America (IDSA) confirmed the indication in the treatment of recurrent CDI with FMT [24].

Alongside well-established indications such as CDI, several studies have found emerging clinical conditions for which FMT may represent a promising alternative to standard therapies. Most evidence comes from inflammatory bowel disease (IBD) studies. Several alterations of gut microbiota has been proposed as factors contributing to the development of the aberrant immunological response in IBD [35], but it is still unclear if the perturbations of microbiota are the cause or consequence of the mucosal inflammation associated to IBD [36]. In particular, ulcerative colitis (UC) is the most suitable IBD model for the study of FMT, considering the characteristics of inflammation of the mucosa and the established role of the microbiota in pathogenesis [37]. To date, a little number of clinical trials have reported promising results, but several concerns suggest to better investigate this potential clinical application [38]. According to a Cochrane systematic review of four clinical trials, the overall remission rate at week 8 was 37% (52/140 UC patients) in patients receiving FMT, compared with 18% (24/137 patients) in those receiving placebo; additionally, clinical response and endoscopic remission improved in patients treated with FMT [39]. However, several factors appear to influence the clinical response in UC patients, as the condition during the manipulation of the feces or the donor selection. For instance, anaerobic conditions during the manipulation of stool were associated with better performance considering clinical remission or steroid free response [40]. Donor selection might be a relevant factor considering that a study reported higher success rates with one particular donor compared with other donors [41]. Furthermore, an emerging relevant indication for FMT was represented by the flare of UC associated with concurrent *C. difficile* over infection. A recent clinical trial, including patients affected by UC or Crohn disease with recurrent CDI, reported that FMT has a curative effect on the recurrence of CDI, but has no apparent beneficial effect on the IBD course [42].

Gut microbiota disturbance was also involved in other gastrointestinal diseases such as irritable bowel syndrome (IBS). A systematic reviews with meta-analysis showed that FMT may be beneficial in IBS [43], but this finding is limited by the small number of patients included and by the relevant differences in the design of the studies. In particular, IBS is triggered by multiple factors, and furthermore, is a heterogeneous condition that may require a selection of the donor in each case. For instance, El-Salhy et al. have recently reported that FMT administered through gastroscope was highly effective in IBS if a well-defined donor was chosen with a normal disbyosis index and favorable specific microbial signature [44].

Furthermore, metabolic and hepatic diseases are also considered emerging indications for FMT. There is great interest towards the modulation of the gut microbiota in metabolic syndrome, as two studies reported promising results in improving peripheral insulin sensitivity [45,46]. Unfortunately, the improvement of metabolic profile was not maintained in the long term, and a recent systematic review including three studies reported the absence of significant benefits from FMT in metabolic syndrome [47]. Thus, further studies to clarify the feasibility of this approach in metabolic disorders are needed. Furthermore, FMT was able to reverse encephalopathy derived from disturbed gut-brain axis in patients with liver chronic disease, two clinical studies shown promising results in this field of application [48,49].

FMT was also proposed in the treatment of several other clinical conditions, but evidence is limited and results were reported by small studies; thus, the application is limited to clinical studies and selected cases. For instance, FMT was reported as effective in the decolonization of patients carrier of multi-drug resistant organism [50], in reducing symptoms in autism spectrum disorders [51], or in reliving symptoms and increasing progression free survival in graft versus host disease after hematopoietic stem cell transplant [52].

## 3. Selection and Screening of Stool Donors

Donor selection represents a fundamental challenge in view of the implementation of FMT programs worldwide. To date, there is a broad debate regarding the preference of donor selection, whether the stool donor should be known to the patient or whether it is preferable to use feces from unrelated donor. Moreover, in the case of non-related donor, fecal material could be banked at dedicated structures that provide support to the hospital that will perform FMT [53].

In particular, the ideal stool donor should be a healthy volunteer, without risk factors for infectious or other chronic diseases, and who is willing to “donate” frequently if needed. Unfortunately, although the conditions do not seem too selective, it is not always easy to identify an adequate number of donors to meet the needs of the FMT program. Indeed, data from large stool bank suggest high rates of donor drop out due to high commitment required [54]; furthermore, physicians often give up FMT because of the complexity and costs of screening [55]. Consequently, to solve these problems, it would be appropriate to implement the undirected donor selection program. Hence, the related donors should be only limited in cases of patient preference. Indeed, undirected donors reduced the likelihood of confidentiality concerns, and then, they are essential for the implementation of stool banking in consideration of easy availability, traceability, and reduction of screening expenses [56].

The screening of potential donors consist in two key landmarks, the preliminary interview and the laboratory testing [13]. A preliminary interview is usually performed by a structured questionnaire that investigated several risk factors to minimize the risk of transferring infections or adverse gut microbiota profile. In particular, the medical interview screen potential donors inquiring about the use of drugs that can alter gut microbiota, known history or risk behaviors for infectious disease, and for disorders potentially associated with the disruption of gut microbiota. The schedule of questions reported in this review (Table 1) includes the most frequently investigated features in leading FMT centers. Obviously, this draft of interview is not mandatory, but can be adapted to the socio-cultural context of potential donors. For example, it would be advisable to carefully investigate the eating habits of potential donors from country where the consumption of raw meat and fish is widespread, thereby increasing the risk of transmission of enteric pathogens, or who eat exotic animals that are potential carriers of unknown pathogens; or seasonal habits that increase the risk to get infected with intestinal pathogens (e.g., summer holidays and risk of sea food of poor quality). These examples allow to understand how the aim of the interview is to early intercept potential risks of pathogen transmission; thus, each center should adapt the medical interview to its socio-cultural context to make it more efficient.

The optimal donor correspond at young individual (preferably < 50 years, as suggested by a panel of experts [13] taking into account that increasing age has been associated with altered gut microbiota composition [57]; moreover, aged microbiota could have a negative effect contributing to the inflammatory state of the recipient [58]), although is important to exclude candidates with personal history of malignancies or autoimmune disease [13]. Moreover, there are concerns regarding the exclusion of healthcare workers considering the supposed increased risk of colonization by antibiotic-resistant bacteria; however, available data suggest a low prevalence in this population [59].

Potential donors who have a permissive medical history must undergo to blood and fecal examination to exclude infective disease transmittable trough fecal transfer [13]. The tests may change between the various protocols, but there are some mandatory examinations (Table 2).

In fact, blood testing should include complete blood cell count, liver enzyme, creatinine, and C-reactive protein to check overall clinical condition, serology for Hepatitis virus, and Human immunodeficiency virus (HIV). Furthermore, blood tests can be considered in case of anomalies of the first round of laboratory tests, endemic spread of some pathogens, emergence of new pathogens or selected cases of recipients (e.g., immunosuppressed). In particular, there is debate about the usefulness of serology for EBV and CMV, as the high prevalence of prior exposure among adult individuals weakens the diagnostic power of this approach, limiting the clinical utility to IgM CMV in donors dedicated to immunosuppressed recipients. Of course, it is not appropriate to exclude subjects with prior exposure to EBV or CMV from the donation because of the unlikely risk of transmission, unless clinical or laboratory suspicion of reactivation. Finally, the candidates could be considered for testing the serology for nematodes, based on social and geographical features and tests availability [13].

Stool testing should include common enteric pathogens, *Clostridium difficile*, fecal parasites, and *Helicobacter pylori* antigen (this last exam only for upper route of FMT delivery). Enteric pathogens, which must also be investigated in asymptomatic subjects, should be detected with conventional methods (culture, microscopy, or antigen test) and/or with molecular diagnosis (PCR-based panels) that have shown a high specificity and sensitivity compared to conventional methods in rapid detection of pathogens [60]. Furthermore, it is mandatory to test all fecal samples for antibiotic-resistant bacteria (including meticillin-resistant *Staphylococcus aureus* (MRSA), vancomycin-resistant Enterococci (VRE), extended-spectrum β-lactamase-producing Enterobacteriaceae, and carbapenem-resistant Enterobacteriaceae/carbapenemase-producing Enterobacteriaceae), considering the burden of the gastrointestinal carriage in asymptomatic subjects [61,62] and the reporting of some serious adverse events associated to sepsis after FMT [13]. Nowadays, due to the emerging Covid-19 pandemic, a panel of international experts has suggested to include in the tests for Sars-CoV-2 a thorough nasopharyngeal swab and/or RNA detection in stool [63].

Finally, if all blood and fecal tests are negative, the candidate is accepted to become a stool donor. Especially in the fecal bank program, the donor should be available to donate on many occasions over time. For this reason, it is advisable to repeat the screening tests every 8–12 weeks and administer a short questionnaire on the same day of the donation to check for any recent-onset harmful events.

In this paragraph we have reported the general rules to select and to screen potential donor for FMT, mainly to treat CDI that is cured by the restorative effect of fecal transfer on gut microbiota. However, for other clinical indications, which find their rationale in the modification of metabolic and inflammatory pathways mediated by gut microbiota, it would be appropriate to identify a specific donor for each case. This issue will be discussed later.

## 4. Selection of the Optimal Donor for FMT to Treat Specific Disorders

The correct recruitment of healthy donors is essential for a standardized and safe FMT procedure [11,13]. FMT is considered a safe procedure; however, mild adverse effects attributable to FMT are reported in about one third of the recipients, such as self-limiting abdominal discomfort or changes of bowel habits, and unfortunately, about 2–6% of patients experienced serious adverse events, such as infection, relapse of pre-existing disease, or death [64]. Moreover, the difficulty of selecting the appropriate candidates is increasing due to emerging concerns, as the possibility of transmission of putative procarcinogenic bacteria [65] or the potential risk of serious life threatening infections with multi-drug resistant organisms after FMT [66]. Moreover, recent evidences showed that the efficacy of FMT in recurrent CDI treatment, in clinical trials and in other healthcare settings seems to be linked to different variables, such as the delivery methods of fecal infusate, the bowel preparation, the number of infusion, the disease severity, and in particular to the microbial diversity and composition of the transplanted stools [32,44,67]. Since the idea that the success rate of FMT could be related to the gut microbiota or other features of the donor, the term “super-donors” has been introduced to indicate the ideal individuals whose stools could ensure a better outcome for recipients compared to others fecal donations [68]. Therefore, assuming that dysbiosis-related disorders have been associated to different imbalanced microbial signatures [15], in order to restore the eubiosis, it is reasonable to assume that reaching the correct donor-recipient match with targeted FMT based on specific microbial disturbances might be the key to improve FMT response. Accumulating evidence strengthens this hypothesis, leading to discard the concept of “one stool fits all” and to search an optimal donor [68], as in other organ transplantation procedures [69].

### 4.1. Clostridium Difficile Infection

The research of the ideal donor in recurrent CDI is obviously a widely debated topic of study. For example, one study identified the optimal donor among nine healthy vegetarian or vegan candidates, selecting the candidate who had a balanced Bacteroidetes/Firmicutes ratio, the highest alpha diversity among screened individuals, and high butyrate concentration. After 10 weeks from a single or multiple FMT, none of the 10 patients experienced CDI recurrence [70]. Of interest, the gut virome may also play a role in CDI treatment [71]. Indeed, enteric virome alterations marked by an increase in the abundance of *Caudovirales*, together with a decreased *Caudovirales* diversity, richness, and evenness, have been reported in patients with CDI. Moreover, CD eradication was associated with the colonization of a higher abundance of donor-derived *Caudovirales* contigs detected during follow up. These findings could possibly explain why bacterial fecal filtrate infusion resulted in effective treatment of CDI [72], and shifted the attention on the importance of the bacteriophages and on the potential role of selecting donors on the basis of their gut virome. Finally, some authors reported that selecting specific enteric bacterial strains with bacterial cultures from healthy donors to prepare a stool substitute blend might be a winning strategy to cure recurrent and antibiotic-resistant *C. difficile* colitis [73,74]. However, it is likely that the relevant impact on FMT success in CDI depends on the transfer of a complete fecal microbiome rather than specific bacterial strains; moreover, the promising results reported by the study that transfer the fecal filtrate alone suggest a predominant role for bacteriophages rather than for the specific relative abundance pattern of the gut microbiota of donor, shifting the central role from bacteria to viruses in the therapeutic challenge of FMT in CDI; however, these data are still preliminary and need to be confirmed by further studies.

### 4.2. Inflammatory Bowel Disease

Many studies analyzed the microbial profile of donors and tried to relate it with clinical and laboratory outcomes in patients with IBD. Clinical outcomes and immunological changes after FMT in patients with IBD were significantly related to the variations of several specific strains in recipients of fecal microbiota [75]. For instance, intensive FMT in UC patients were associated with negative outcomes in case of abundance of *Fusobacterium spp* and *Sutterella spp* in recipients’ fecal microbiota after the FMT [76]. Furthermore, a study that involved refractory UC patients reported that pre-treatment with antibiotic plus repeated FMTs using fecal material from donor with a high bacterial richness and high relative abundance of *Akkermansia muciniphila*, unclassified Ruminococcaceae, and *Ruminococcus spp*. was more likely to induce remission compared to antibiotics alone [67]. As also described in other studies [41,77,78], it is plausible that choosing donors based on their taxonomic composition, in particular low or high abundance of specific strains, might reflect the possibility for future trials in IBD. For this purpose, methods aimed at preventing an inflammatory response of the recipient’s intestinal immune system by selecting compatible donors on their microbial profiles are under study [79]. Furthermore, the gut virome could represent a potential marker for FMT response in UC patients. In particular, results from a small case series reported that FMT responders already presented, before undergoing to FMT, a significantly lower eukaryotic viral richness than non-responders. Moreover, the richness of donor virome was not associated with the FMT outcome, as instead proposed for bacteria [80].

### 4.3. Other Emerging Indications

Several preclinical and clinical studies supported the rationale for donor selection based on gut microbial profile in other disorders associated to gut dysbiosis. Indeed, in the field of anti-cancer treatment, it has been reported that microbiota can influence chemotherapy response [81]. Preclinical studies found a clinical improvement in mouse models of melanoma on anti-PD-1 therapy that received FMT from donors with a melanoma “responder-like” microbial signature (with high alpha diversity and abundance of Ruminococcaceae, *Faecalibacterium*, *Bifidobacterium longum*, *Collinsella aerofaciens*, and *Enterococcus faecium*) when compared to mice that received “non responder-like” microbiome (characterized by low microbial diversity and high relative abundance of Bacteroidales) [82,83]. Nevertheless, trials on humans, testing the effect of FMT in increasing the response to cancer therapies, are still in progress [84].

Recently, a randomized placebo-controlled trial of FMT in IBS reported that the abundance of *Streptococcus*, *Dorea*, *Lactobacillus*, and *Ruminococcaceae* spp in the donor microbiota was associated with efficacy in relieving IBS symptoms [44]. Interestingly, a small open-label clinical trial evaluated the impact of prolonged FMT with antibiotic pre-treatment in children with autism; authors reported a decrease of gastrointestinal symptoms and an improvement of behavior, together with specific genera increase in recipients (*Bifidobacterium*, *Prevotella*, and *Desulfovibrio*). Conversely, *Prevotella*, and *Desulfovibrio* were more represented in recipients after FMT than in the donor samples, suggesting that unknown factors changed the intestinal ecosystem, making it more hospitable to these strains [85].

Within the context of metabolic diseases, the effect of allogenic FMT post-Roux-en-Y gastric bypass donors was compared with metabolic syndrome donors on glucose metabolism and other parameters in treatment-naïve patients with metabolic syndrome. The authors assessed a decrease of insulin sensitivity in recipients who received FMT from donors with metabolic syndrome compared with using post-surgical donors. Moreover, they identified several microbial OTUs possibly predictive of metabolic response, suggesting a microbiota-related transmissible mechanism of insulin resistance [86]. Similarly, another study reported a significant increase in insulin sensitivity, together with altered microbiota composition, in patients with metabolic syndrome who received allogenic FMT from lean donors compared to those who underwent autologous FMT [46].

To date, these results appear promising but partially controversial; thus, findings need to be confirmed with stronger evidence and by standardized clinical trial. Further research is needed to identify the favorable microbial signature of donor or other ideal features in disease-specific settings.

## 5. Conclusions and Future Perspectives

In this review, the stool donor screening process has been described, and recent evidence has been reported that try to identify the optimal donor for each clinical condition (Figure S1).

To date, the clinical characteristics of the donor are well defined; in particular, they are recommended to be a healthy volunteer with a balanced lifestyle, without chronic diseases or family history of metabolic diseases or cancer, and defined laboratory exams must certify the current absence of disease. However, identification of the ideal donor through the microbiological typing of the stool is currently not suitable. First of all, understanding the role of the intestinal microbiota in each chronic disease is an indispensable condition before hypothesizing a personalized approach through FMT. In fact, while the restorative mechanism of FMT in recurrent CDI is now understood, many aspects still need to be understood regarding the treatment of other chronic conditions. Interesting evidence has been reported regarding dysbiosis in IBD or in other chronic conditions, but the contrasting results reported in clinical trials of FMT could be justified by the choice of unsuitable donors. The identification of the microbiological characteristics of the ideal donor for each disease appears to be an achievable goal but still far from being accomplished due to the lack of clinical studies. The current evidence is still limited and insufficient for explaining and resolving the complexity of the interaction between the intestinal barrier and its role in gut-related chronic diseases. However, further studies need to be designed to confirm the encouraging results that have been reported in recent years. In particular, it will be necessary to type the fecal microbiota of the donor and the recipient, and to understand how environmental factors, such as diet, or individual features may benefit (or not) the clinical response to FMT. Understanding the microbial characteristics of the optimal donor, in particular if they are modifiable through lifestyle changes or pharmacological measures, could increase the therapeutic potential of FMT.

## Figures and Tables

**Table 1:** Preliminary interview to select donors.

Preliminary Interview – Medical History
**Drugs that can alter gut microbiota**
Use in the last three months of: ▪ Antimicrobial drugs ▪ Immunosuppressant agents ▪ Chemotherapy Daily use for over three months: ▪ Proton pump inhibitors
**Disorders potentially associated with the disruption of gut microbiota:**
▪ Personal history of chronic gastrointestinal disease, including functional gastrointestinal disorders; inflammatory bowel disease; celiac disease; other chronic gastroenterological diseases or recent abnormal gastrointestinal symptoms (e.g., diarrhea, hematochezia, etc.) ▪ Personal history of cancer, including gastrointestinal cancers or polyposis syndrome, and first-degree family history of premature colon cancer ▪ Personal history of systemic autoimmune disorders ▪ Obesity (body mass index > 30) and/or metabolic syndrome/diabetes ▪ Personal history of neurological/neurodegenerative disorders ▪ Personal history of psychiatric/neurodevelopmental conditions
**Know history or risk behaviors for infectious disease**
▪ History of HIV, hepatitis B or C viruses, syphilis, human T-lymphotropic virus I and II ▪ Current systemic infection ▪ Use of illegal drugs ▪ High-risk sexual behavior ▪ Previous tissue/organ transplant ▪ Recent hospitalization or discharge from long-term care facilities ▪ High-risk travel ▪ Needle stick accident in the last six months ▪ Body tattoo, piercing, earring, acupuncture in the last six months ▪ Enteric pathogen infection in the last two months ▪ Acute gastroenteritis with or without confirmatory test in the last two months ▪ History of vaccination with a live attenuated virus in the last two months

**Table 2:** Donor blood and stool testing.

**Blood testing**
▪ Complete blood cell count ▪ Liver enzyme (Aminotransferases) ▪ Bilirubin ▪ Creatinine ▪ C-reactive protein ▪ Serology for Hepatitis virus (HAV, HBV, HCV, HEV) and Human immunodeficiency virus (HIV)
**Stool testing**
▪ *Clostridium difficile* ▪ *Giardia lamblia, Cryptosporidium* spp, Isospora and Microsporidia ▪ Protozoa and helminths and parasites (including *Blastocystis hominis* and *Dientamoeba fragilis)* ▪ Antibiotic-resistant bacteria ▪ Common enteric pathogens, including *Salmonella*, *Shigella*, *Campylobacter*, shiga toxin-producing *Escherichia coli*, Yersinia, and *Vibrio cholerae* ▪ Norovirus, rotavirus, adenovirus ▪ *Helicobacter pylori* fecal antigen

## Supplementary Materials

The following are available online at https://www.mdpi.com/2077-0383/9/6/1757/s1, Figure S1: Optimal Stool Donor to Treat Specific Disorders.
