# Supplementary figures and images for: Fecal Microbiota Transplantation: Screening and Selection to Choose the Optimal Donor
Source: J Clin Med. 2020 Jun 5;9(6):1757. doi: 10.3390/jcm9061757 (PMC7356099; doi:10.3390/jcm9061757)

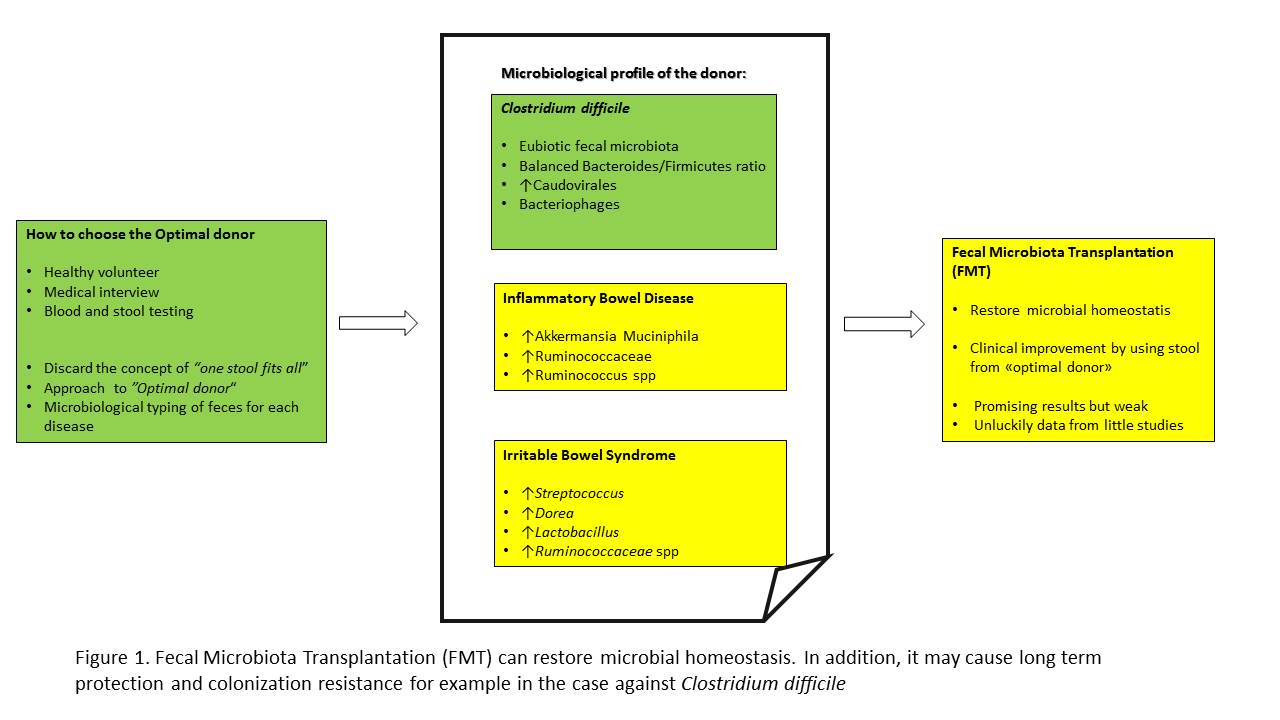

Supplement: Supplementary file 1 [file jcm-09-01757-s001.zip › jcm-802910-supplementary.jpg]
